# Network motifs and hypermotifs in TGFβ-induced epithelial to mesenchymal transition and metastasis

**DOI:** 10.3389/fsysb.2023.1099951

**Published:** 2023-03-03

**Authors:** Gottumukkala Sai Bhavani, Anbumathi Palanisamy

**Affiliations:** Department of Biotechnology, National Institute of Technology Warangal, Telangana, India

**Keywords:** epithelial to mesenchymal transition, metastasis, cancer, C1FFL, hypermotifs, systems biology, motifs, TGFβ

## Abstract

Epithelial to mesenchymal transition (EMT) is a complex, non-linear, dynamic multistep process that plays an integral role in the development of metastatic cancers. A diverse range of signaling molecules, along with their associated pathways, were observed to be involved in promoting EMT and cancer metastasis. Transforming growth factor–β (TGFβ), through its SMAD-dependent and SMAD-independent signaling, orchestrates numerous regulators that converge on key EMT transcription factors (TFs). These TFs further govern the phenotypic transition of cancer cells from epithelial to mesenchymal states. This study explores the TGFβ signaling pathway and its unique network architecture to understand their information processing roles in EMT. Two coherent type 1 feed forward network motifs regulating the expression of SNAIL and N-cadherin were observed. SNAIL, which is one of the crucial regulators of EMT, links both the coherent type 1 feed forward loops (C1FFLs) leading to hypermotif-like structure (Adler and Medzhitov, 2022). Systems modeling and analysis of these motifs and hypermotifs illustrated several interesting emergent information processing roles of the regulators involved. The known roles of these regulators, as described in the literature, were highly correlated with the emergent properties observed. The motifs illustrated persistence detection and noise filtration in regulating the expression of SNAIL and N-cadherin. Along with these system-level properties, the hypermotif architecture also exhibited temporal expression of GLI, SNAIL, ZEB, and N-cadherin. Furthermore, a hypothetical three-layered C1FFL hypermotif was postulated and analyzed. The analysis revealed various interesting system-level properties. However, possible existence of such real biological networks needs further exploration both theoretically and experimentally. Deciphering these network motifs and hypermotifs has provided an additional understanding of the complex biological phenomenon, such as EMT in cancer metastasis.

## Introduction

Cancer metastasis is the development of secondary tumors distant from the primary site. It is a non-linear process involving multiple parallel overlapping routes and includes a variety of cellular mechanisms (Chaffer and Weinberg, 2011; Lambert et al., 2017; Suhail et al., 2019). Epithelial to mesenchymal transition (EMT) is a highly regulated cell developmental program and has been considered a key mechanism of cancer metastasis (Savagner, 2001; Yang and Weinberg, 2008; Kalluri and Weinberg, 2009; Thiery et al., 2009; Nieto et al., 2016; Tripathi et al., 2020). EMT plays an integral role in facilitating the phenotypic transition of adherent epithelial cells into invasive migratory mesenchymal cells (Hay, 1995; Gotzmann et al., 2004; Heerboth et al., 2015). Several studies have revealed the essential roles of numerous signaling pathways in mediating the dynamic, reversible process of EMT and cancer metastasis (Gotzmann et al., 2004; Lo et al., 2007; Thomson et al., 2008; Thiery et al., 2009; Borthwick et al., 2012; Zhang et al., 2013; Zhou et al., 2015; Nieto et al., 2016; Burger et al., 2017). Among many signaling pathways, transforming growth factor–β (TGFβ) signaling and its family of cytokines are well-known regulators of EMT.

The TGFβ superfamily is known to be involved in several cellular processes, such as cell proliferation, differentiation, morphogenesis, and homeostasis. Its implications in EMT have been widely explored in both embryogenesis and tumor development (Massague et al., 2000; Zavadil and Bottinger, 2005; Bierie and Moses, 2006; Massague and Gomis, 2006; Xu et al., 2009; Massague, 2012; Hao et al., 2019). TGFβ, along with its SMAD-dependent and SMAD-independent pathways, orchestrates an elaborate set of regulators in moderating the process of EMT (Attisano and Wrana, 1996; Heldin et al., 1997; Attisano and Wrana, 1998; Miyazono, 2000; Derynck and Zhang, 2003; Massague et al., 2005; Moustakas and Heldin, 2005; Zhang, 2009; Mu et al., 2012; Zhu et al., 2016; Song et al., 2021). These signaling regulators are often involved in complex interactions, leading to the activation of several downstream transcription factors (TFs) that govern the process of EMT in carcinogenesis (Barrallo-Gimeno and Nieto, 2005; De Craene et al., 2005; Lindsey and Langhans, 2014; Weidemüller et al., 2021). For instance, the cross-regulation among TGFβ-stimulated SMAD-dependent and SMAD-independent signaling forms a relay from SMAD to GLI that activates and regulates the expression of the EMT transcription factor SNAIL (Zhang et al., 2018). SNAIL is one of the major EMT transcription factors induced by TGFβ, and it is involved in a broad spectrum of functions, including cell survival, immune regulation, stem cell regulation, and tumor recurrence (Hemavathy et al., 2000; Wu and Zhou, 2010). In recent years, research has focused on identifying the key regulators and characterizing the regulatory circuits involved in EMT.

Decision-making circuits with toggle switch-like responses that lead to multistability during EMT have been widely explored (Lu et al., 2013a; Tian et al., 2013; Huang et al., 2017; Gómez Tejeda Zañudo et al., 2019; Biswas et al., 2022). The core regulatory network explored in such studies comprises of SNAIL:miR-34, ZEB:miR-200 circuits in regulating TGFβ induced EMT (Lu et al., 2013b; Tian et al., 2013; Jia et al., 2017). Dynamic analysis of this network has revealed significant insight into the multistable behavior and existence of hybrid E/M phenotypes (Lu et al., 2013b; Lu et al., 2014; Boareto et al., 2016; Jia et al., 2017; Xin et al., 2020). Apart from this widely studied network structure, there are several network motif-like architectures that govern the process of EMT. These motifs comprise the core EMT regulators, such as SNAIL and ZEB.

**FIGURE 1:**
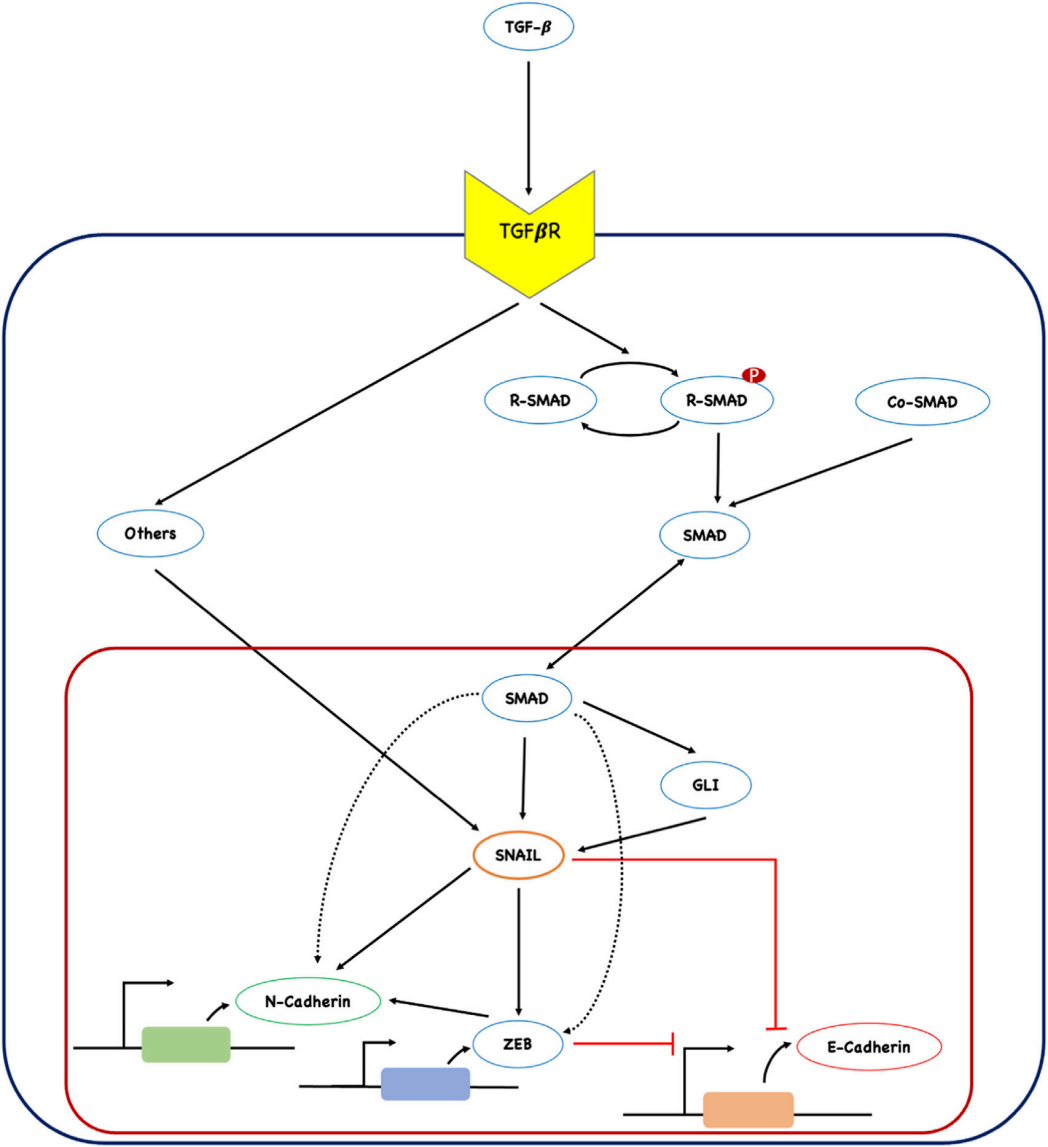
Network of TGFβ-induced epithelial to mesenchymal transition (EMT). The input signal from TGFβ activates both SMAD-dependent and SMAD-independent (represented as ‘others’) signaling, resulting in the regulation of the downstream transcription factor SNAIL. SNAIL further regulates the expression of other regulators involved in the execution of EMT. The network comprises of several network motif like architecture that plays a crucial role in moderating EMT. The bold black arrow represents activation, and the blunt red arrow represents inhibition.

The current work explores the role of TGFβ induced signaling pathway culminating into a set of key regulators of EMT (Figure 1). SMAD, GLI, SNAIL, and ZEB are the key regulators governing the phenotypic markers N-cadherin and E-cadherin in mediating the process of EMT. These regulators are integral to the complex interconnected networks involved in various cellular processes, such as cell fate decisions during embryonic development, cellular reprogramming, and phenotypic switching (Massague et al., 2005; Wu and Zhou, 2010; Macneil and Walhout, 2011; Gheldof et al., 2012; Aberger and Altaba, 2014; Singh et al., 2014; van Roy, 2014). The network assembled in this study comprised several unexplored coherent type 1 feed forward loops (C1FFLs) and hypermotifs. The key biological attributes of EMT can be manifested by these network-level interactions. In this work, embedded feed-forward loops (FFLs) and hypermotifs were characterized using dynamics modeling and analysis. The biological relevance of such network motifs was also explored. The observed results illuminate the role of several underlying feed-forward motif architectures in the regulation of EMT.

## Results

### TGFβ-induced epithelial to mesenchymal transition network

A TGFβ-induced EMT network (Figure 1) was assembled by reviewing the literature. One of the major EMT transcription factors, SNAIL, was found to be at the heart of the network induced by TGFβ signaling. TGFβ regulates the expression of SNAIL through both SMAD-dependent and SMAD-independent pathways (Derynck and Zhang, 2003; Smith et al., 2009). Along its SMAD-dependent pathway, TGFβ ligands activate their receptors through phosphorylation. Activated receptors phosphorylate R-SMADs, promoting R-SMAD: Co-SMAD complex formation for nuclear entry (Heldin et al., 1997; Attisano and Wrana, 2002). In this work, the R-SMAD: Co-SMAD complex is represented as SMAD, as shown in Figure 1. TGFβ has also been shown to regulate the expression of GLI, a zinc finger protein and effector of hedgehog signaling, through the transcriptional activation of SMAD (Javelaud et al., 2011). GLI facilitates the degradation of epithelial cells through regulating the expression of SNAIL (Dennler et al., 2007).

TGFβ-induced SMAD is known to transcriptionally regulate the expression of other major EMT transcription factors, including SNAIL, ZEB, and mesenchymal marker N-cadherin (Shirakihara et al., 2007; Gregory et al., 2011; Gheldof et al., 2012; Yang et al., 2015). SNAIL and ZEB, regulated by the TGFβ/SMAD signaling axis, positively regulate the expression of the mesenchymal gene N-cadherin and negatively regulate the expression of the epithelial gene E-cadherin during EMT (Batlle et al., 2000; Cano et al., 2000; Chen et al., 2016). Apart from the TGFβ/SMAD signaling pathway, several SMAD-independent pathways, such as PI3K/Akt, MAPK, ERK, and NF-kB, are known to impact the expression of SNAIL (Barrallo-Gimeno and Nieto, 2005; De Craene et al., 2005; Giannelli et al., 2005; Moustakas and Heldin, 2005). In this work, these pathways are cumulatively represented as ‘others’ (Figure 1). TGFβ-induced SMAD-dependent and SMAD-independent pathways converge in the regulation of the network of EMT transcription factors and genes that execute EMT during cancer metastasis.

### Network motifs involved in TGFβ-induced EMT

**FIGURE 2:**
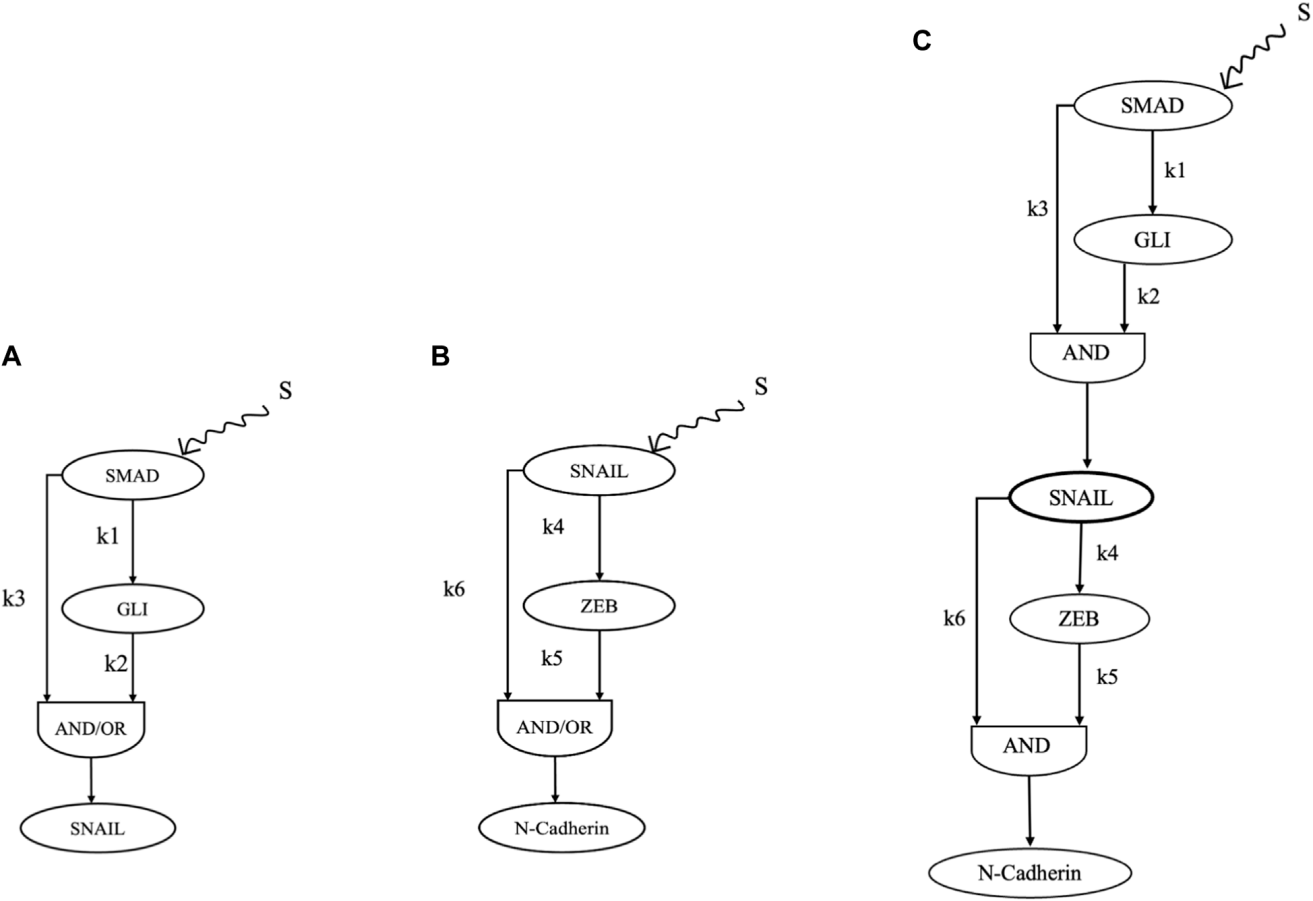
Network motifs from the assembled network that critically regulate the process of EMT induced by TGF β. **(A)** C1FFL regulation of SNAIL. **(B)** C1FFL regulation of N-cadherin. **(C)** Combination of C1FFLs in regulating the expression of N-cadherin.

The TGFβ-induced SMAD interaction network (Figure 1) assembled for this study possessed two coherent type 1 feed-forward loops (Figures 2A, B). Both C1FFLs were organized in a sequential layer-like architecture regulating the final expression of N-cadherin (Figure 2C). SNAIL was directly regulated by TGFβ-induced SMAD and indirectly regulated through GLI in a C1FFL manner (Figure 2A). SNAIL regulated the mesenchymal gene N-cadherin both directly and indirectly through ZEB, which resulted in a C1FFL motif (Figure 2B). Furthermore, these C1FFLs were observed to be structured in a cascade or layer like architecture in which the output from the first motif (SNAIL) is the input for the second motif in regulating the expression of N-cadherin (Figure 2C). Thus, SNAIL functions as an intermediate regulator by integrating the information. The observed network motifs connected through SNAIL resulted in a hypermotif-like (Adler and Medzhitov, 2022) architecture.

### Modeling and analysis of C1FFLs

**FIGURE 3:**
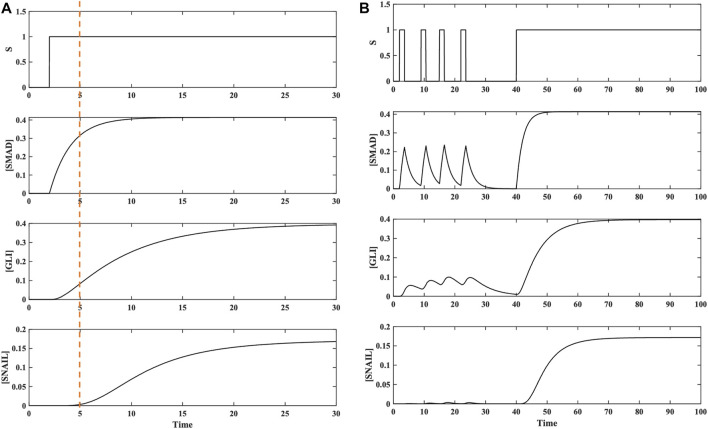
Dynamics of TGFβ-induced activation of SNAIL in a coherent type 1 feed-forward regulation carried out directly by SMAD and indirectly through GLI. **(A)** Dynamics of C1FFL with respect to AND regulatory logic for input stimulus S. A delay in the ON step was observed for SNAIL transcriptional activation. The delay time was observed to be ∼2 timesteps after the ON step. No delay was observed during the OFF process when the stimulus S was withdrawn. **(B)** Dynamics of the C1FFL in response to brief pulsatile stimulus S. When pulse stimulus in brief intervals was given, the C1FFL motif was observed to act as a Noise Filter and act as a persistent detector when receiving a continuous stimulus, which is reflected in the expression of SNAIL.

**FIGURE 4:**
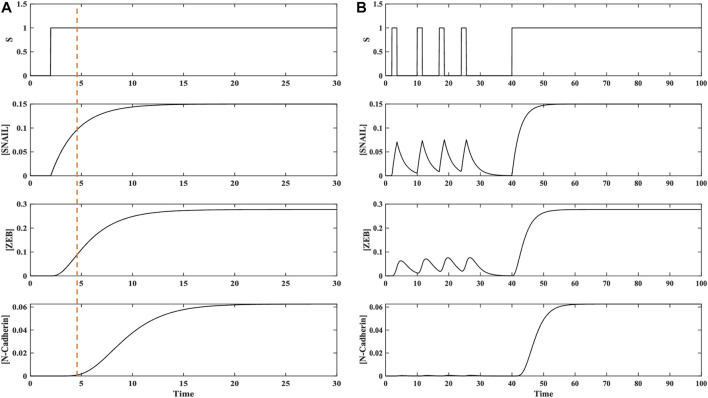
Regulation of mesenchymal gene N-cadherin by SNAIL and ZEB induced by TGFβ in a C1FFL manner. **(A)** Dynamics of C1FFL with respect to AND regulatory logic for input stimulus S. A delay in the ON step was observed for N-cadherin transcriptional activation. The delay time was observed to be ∼2 timesteps after the ON step. No delay was observed during the OFF step when the stimulus S was withdrawn. **(B)** Dynamics of the C1FFL for brief pulsatile stimulus S. When a pulse stimulus in brief intervals was given, the C1FFL motif was observed to act as a noise filter; and act as a persistent detector for a continuous stimulus, which is reflected in the expression of N-cadherin.

To explore the role of the C1FFLs in moderating the process of EMT and cancer progression, dynamic modeling, simulation, and analysis were performed. The C1FFLs, individually and in combination in a layered architecture, referred to as a hypermotif (Adler and Medzhitov, 2022), were extensively characterized. For an input stimulus S received from TGFβ, the C1FFLs (Figures 2A, B) were characterized reliant on the position of SNAIL with both AND, OR logic. OR logic-like activation of both the C1FFLs resulted in a delayed OFF step (Supplementary File 1, Supplementary Figures S1, S2), which was not relatable to the observed biological roles of these regulators. However, when utilizing AND logic-like activation, both the C1FFLs individually exhibited the system-level properties of sign-sensitive delay, noise filtration, and persistence detection in modulating the EMT of cancer cells (Figures 3, 4). EMT occurs as a result of the combined effects of the upstream regulators SMAD and GLI influencing the expression of SNAIL (Figure 2A), which further disrupts the epithelial phenotype through ZEB and N-cadherin (Figure 2B).

### Regulation of N-cadherin expression

Regulation of the mesenchymal gene N-cadherin comprises two C1FFLs in sequential layers (Figure 2C), which were individually explored to assess their biological roles, as described in the previous section. Furthermore, to understand the interconnected dynamics of these layered C1FFLs, the combined network/hypermotif shown in Figure 2C was modeled and analyzed utilizing the continuous ODE modeling. This module received input stimulus from TGFβ (S), the end response elicited is the expression of N-cadherin. N-cadherin is one of the major mesenchymal markers observed to play a crucial role in cell adherent and migratory properties.

**FIGURE 5:**
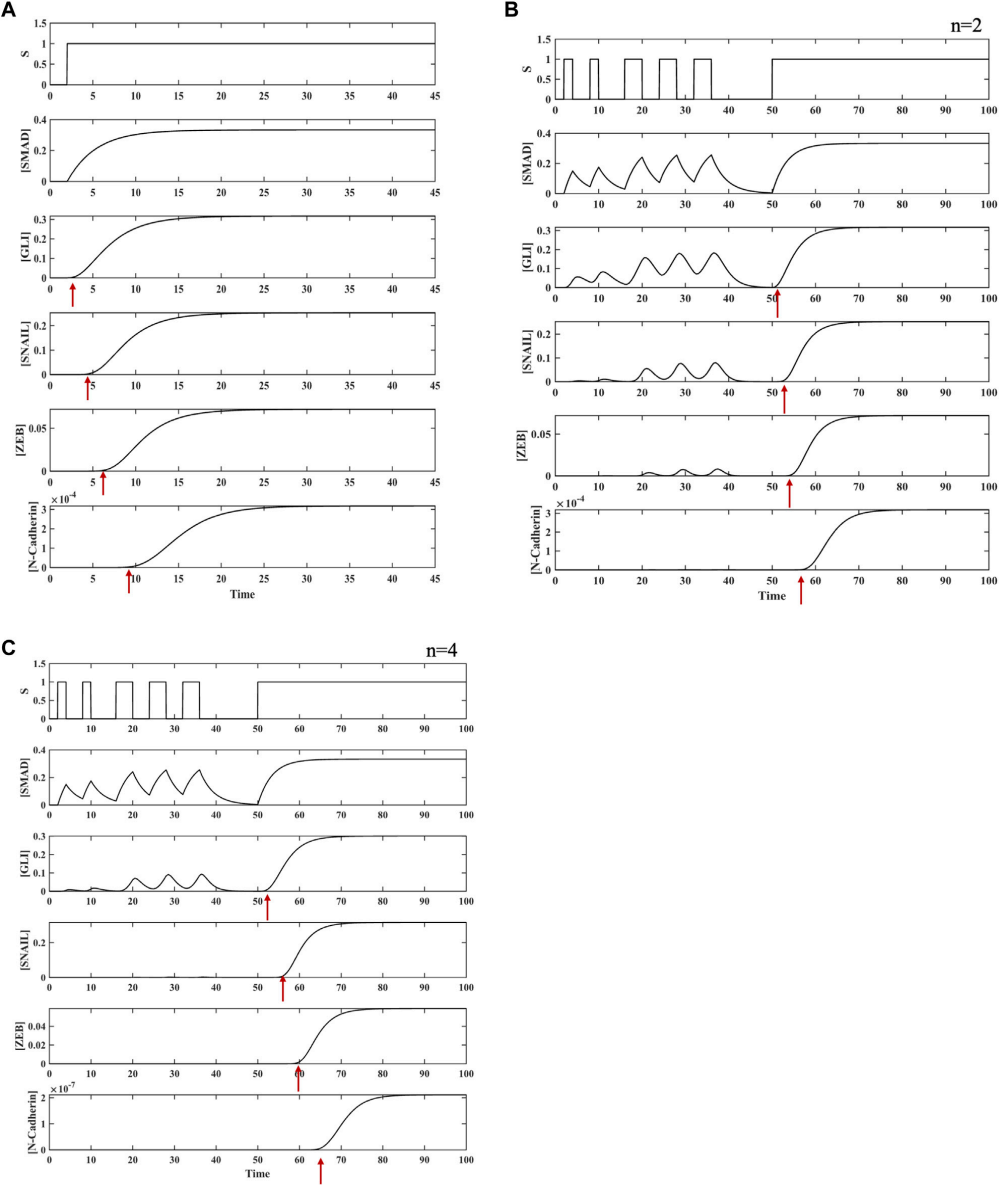
Combination of two C1FFLs (hypermotif) regulating N-cadherin expression. **(A)** Dynamic activation of TGFβ-induced EMT regulators in a hypermotif-like architecture. Temporal activation of GLI, SNAIL, ZEB, and N-cadherin was observed with delay relative to their upstream regulators. **(B)** Emergent properties, such as noise filtration (pulsatile stimulus) and persistence detection, (continuous stimulus) were observed. **(C)** Dynamics of the hypermotif for the Hill coefficient *n* = 4 demonstrate that larger n contributes for potent noise filter and a persistent detector in regulating N-cadherin.

In the presence of stimulus S, SMAD and GLI regulates the expression of SNAIL. SNAIL further relays the signal to ZEB in regulating N-cadherin in an AND logic dependent manner. Based on the inferences from the individual motif analysis we are exclusively considering only AND logic like activation of the layered network/hypermotif. Modeling and analysis of this hypermotif displayed temporal activation of SNAIL, ZEB, and N-cadherin (Figure 5A). When SNAIL is getting regulated through SMAD and GLI it functions like a signal integrator, relaying the information to the downstream regulators ZEB and N-cadherin (Figure 5A). ZEB further processes the signal from SNAIL in regulating the EMT by activating mesenchymal gene N-cadherin. This expression of N-cadherin was observed to be temporally delayed relative to SNAIL activation because it requires both the regulators SNAIL and ZEB. N-cadherin expression leads to enhanced invasive potential, stemness with migratory behavior of cancer cells.

Pulsatile input stimulus S was provided to the hypermotif to further explore the influence of the type of stimulus on the expression of N-cadherin. Its expression was observed to respond only to persistent stimuli (Figure 5B). The sequential cascade with coherent type 1 network architecture, which requires both SMAD and GLI binding in the first layer and both SNAIL and ZEB binding in the second layer, filtered out the short fluctuations in the upstream signals (Figure 5B). Furthermore, to assess the effect of cooperativity between the regulators on system-level properties, the Hill coefficient was varied (*n* = 2–4). For *n* = 4, an enhanced filtering effect was observed to regulate the expression of N-cadherin (Figure 5C, Supplementary File 1, Supplementary Figure S3). Thus, the C1FFL modules in a layered architecture, also known as a hypermotif, functions as a persistence detector and potent noise filter with AND logic-like regulation to avoid spurious activation of N-cadherin.

### Three-layered generic cascade of C1FFLs

**FIGURE 6:**
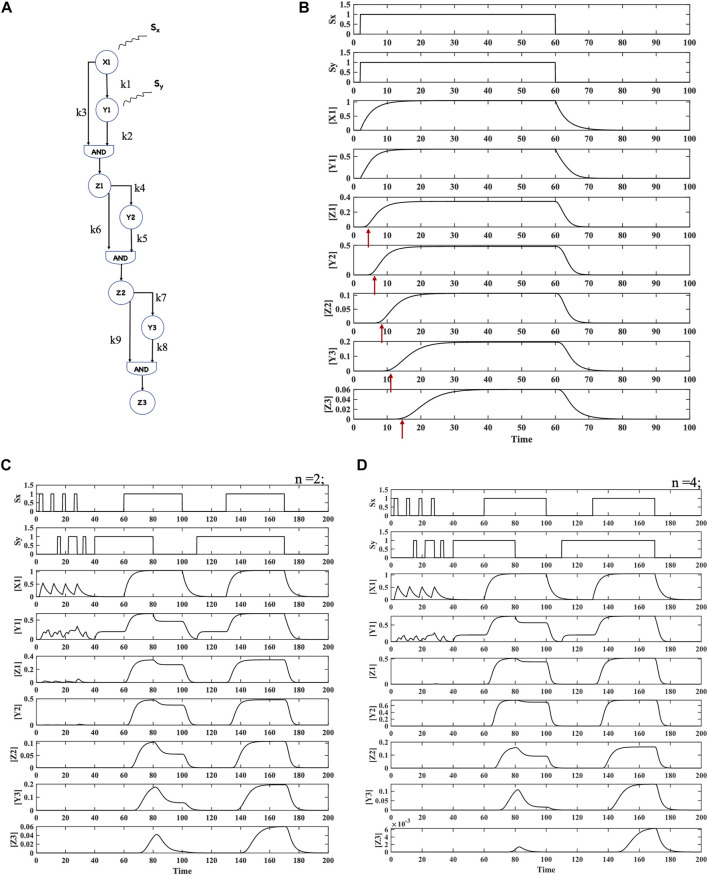
Hypothesized combination of three C1FFLs (hypermotif) in layers and the resulting dynamics. **(A)** Three-layered coherent type 1 feed-forward network postulated with AND logic-like activation. **(B)** Dynamics of the hypermotif for stimuli S_x_ and S_y_. A delay of ∼2, 5, and 11 time steps was observed for the regulators Z_1,_ Z_2_, and Z_3_, respectively. No response was observed when the stimulus was withdrawn. **(C**,**D)** Dynamics of the hypermotif for Hill coefficients *n* = 2 and *n* = 4, respectively. For both n values, emergent noise filtration and persistence and coincidence detection were observed. However, the higher Hill coefficient favored effective noise refinement, indicating a possible role of cooperativity.

Motivated by the unique insights gained from the two-layered C1FFL hypermotif, a three-layered C1FFL hypermotif was designed and analyzed. The three-layered hypermotif included a C1FFL at each layer of the cascade (Figure 6A **)**. The hypothetical network received two input stimuli, S_x_ and S_y_, and provided a single end response Z_3_. There were two intermediate nodes, Z_1_ and Z_2_, which integrates the layered cascades.

ODE-based modeling, simulations, and analyses were performed to assess the response of the network/hypermotif to various input combinations. The signaling was initiated by the activation of X_1_ through the stimulus S_x_ and activation of Y_1_ through the stimulus S_y_. X_1_ together with Y_1_ acts in an AND logic C1FFL manner in regulating the output Z_1_ (Figure 6A). The results for this hypermotif generated an expression of Z_1_ with a delay (Figure 6B), which was relayed as input along with Y_2_ to the subsequent layer regulating the expression of Z_2_. Expression of Z_2_ was delayed relative to Z_1_. Z_2_ was relayed as an input along with Y_3_ to the subsequent layer for regulation of Z_3_ expression. Delayed expression of Z_3_ relative to the upstream regulators resulted in temporally regulated expression (Figure 6B). Thus, the layered combination of C1FFLs/hypermotifs were observed to relay signal leading to temporal expression at various levels.

In the presence of stimulus S_x_, when stimulus S_y_ was removed, the network elicited a temporal response with a longer delay (Supplementary File 1, Supplementary Figure S5). However, in the absence of stimulus S_x_ and in the presence of stimulus S_y_, the circuit did not elicit any response (Supplementary File 1, Supplementary Figure S6). Furthermore, a pulsatile input stimulus for each layer was provided to the C1FFL cascade to measure the hypermotif response. For short pulses of S_x_ and S_y_, the C1FFL cascade/hypermotif did not elicit a response of Z_3_, clearly indicating a functional noise filter (Figure 6C). Along with noise filtration, the network also functioned as a persistence and coincidence detector (Figure 6C). Furthermore, to determine the effect of the Hill coefficient (*n*), simulations were performed with n varying from 2 to 4. A Hill coefficient of 4 elicited a stronger noise filtering behavior along with persistent and coincidence detection (Figure 6D, Supplementary File 1, Supplementary Figure S4).

### Parameter sensitivity analysis of motifs and hypermotifs

**FIGURE 7:**
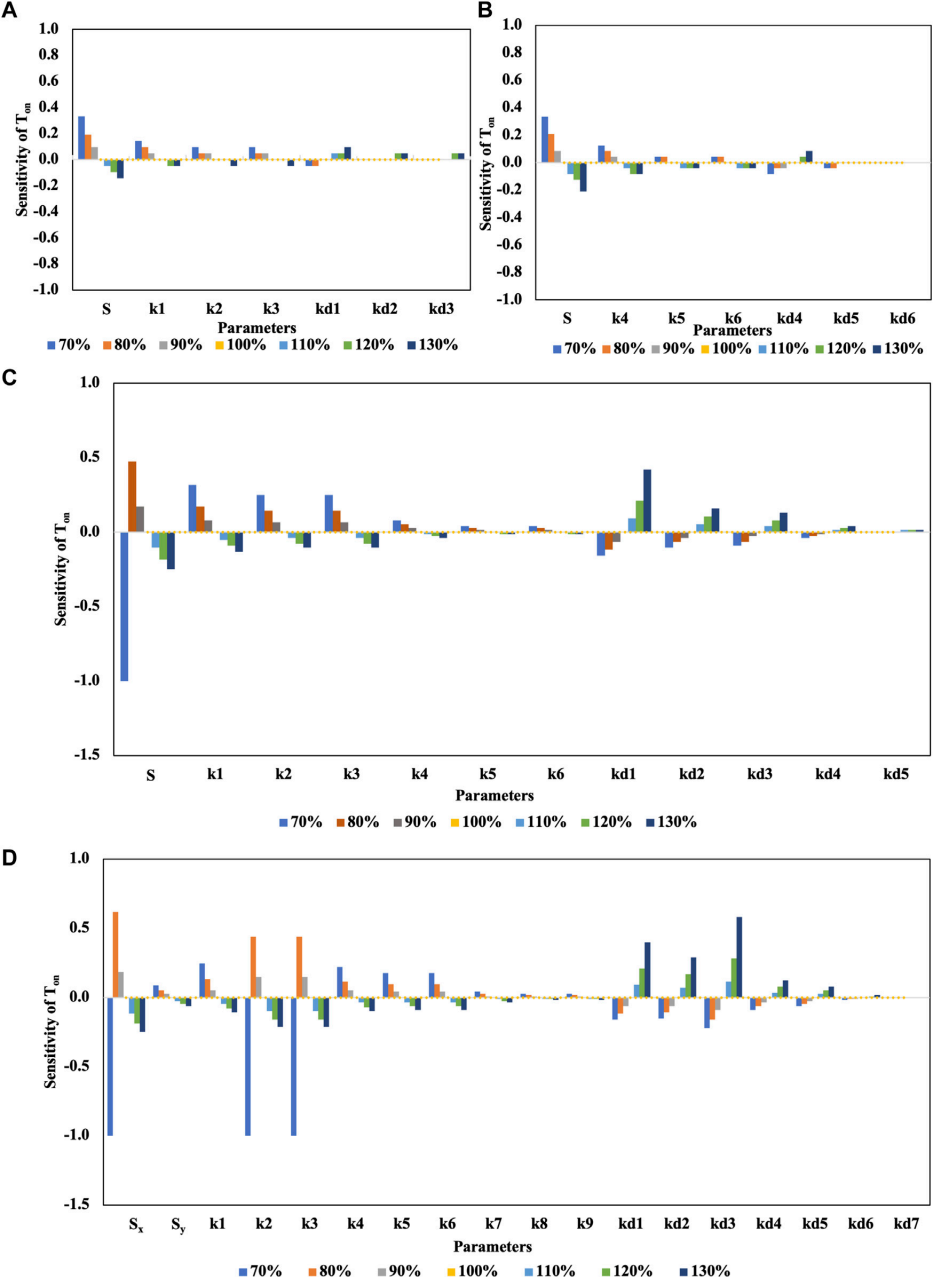
Local parameter sensitivity analysis of motifs and hypermotifs. The bar graphs show the relative sensitivity metrics of T_on_ for individual parameter variations of ±30% from their nominal values. **(A)** Sensitivity plot for SNAIL regulated by SMAD and GLI in a C1FFL. **(B)** Sensitivity plot for N-cadherin regulated by SNAIL and ZEB in a C1FFL. **(C)** Sensitivity plot for N-cadherin regulated by a hypermotif. **(D)** Sensitivity plot for Z_3_ of the hypothesized hypermotif.

The impact of variation in the model parameters (±30%) on T_on_ is shown in Figure 7. While the estimated T_on_ for motifs in Figures 2A, B are insensitive to certain parameter variations (k2, k3, kd1, kd2, kd3, k5, k6, kd4, kd5, and kd6), it is highly sensitive to parameters associated with synthesis rates of SMAD and SNAIL (Figure 7A, B). T_on_ decreased with an increase in TGFβ-induced activation of SMAD (S) and increased with a decrease in TGFβ-induced activation of SMAD (S) (Figure 7A). Furthermore, the C1FFL shown in Figure 2B was sensitive to changes in the activation rate of SNAIL. T_on_ decreases with an increase in SNAIL levels (S) and increases with a decrease in SNAIL levels (S) towards regulating N-Cadherin (Figure 7B). Local sensitivity analysis performed on the hypermotif regulating N-cadherin (Figure 2C) revealed that T_on_ was highly sensitive to parameter variations associated with the top-layer motif. The parameters associated with SMAD, GLI, and SNAIL (S, k1, k2, k3, kd1, kd2, and kd3) affected the regulation of N-cadherin. The T_on_ of N-cadherin decreased with an increase in activation parameters and with a decrease in degradation parameters (Figure 7C). Conversely, T_on_ of N-cadherin increased with a decrease in activation parameters and with an increase in degradation parameters. Thus, parameters associated with the SMAD, GLI, and SNAIL regulators exhibited stronger sensitivity and are likely to play a key role in controlling the dynamics of EMT. The local sensitivity analysis of the hypothesized hypermotif (Figure 6A) revealed that the T_on_ of Z_3_ was sensitive to the parameters associated with the foremost layers of the hypermotif (Figure 7D). Variation in parameters associated with C1FFL in the first layer (X_1_, Y_1_, and Z_1_) impacted T_on_ in the regulation of the end response regulator Z_3_. Increased activation of X_1_, Y_1_, and Z_1_ resulted in early activation of Z_3_, while curtailed expression of X_1_, Y_1_, and Z_1_ resulted in delayed activation of Z_3_ (Figure 7D). The local parameter sensitivity analysis demonstrated an impact only on the activation time (T_on_) of the end response regulator and no change in the overall responses of the motifs and hypermotifs.

## Discussion

This assembled network demonstrates the existence of interesting network architecture among various regulators of TGFβ-induced EMT. TFs are the key regulators of cellular processes, and they serve as a link between signaling pathways and gene regulation (Mangan and Alon, 2003; Weidemüller et al., 2021). TFs play a significant role as interacting partners and information processors, and they form several recurring patterns, such as autoregulatory loops, feedback loops, feed-forward loops, and cascades, which are commonly referred to as network motifs (Shen-Orr et al., 2002). Network motifs are the building blocks of complex biological networks for computing behavioral dynamics (Milo et al., 2002; Adler and Medzhitov, 2022). System-level dynamic properties of network motifs, such as FFLs and their role in noise regulation, coincidence and persistence detectors, have been previously explored (Mangan and Alon, 2003; Ferrell, 2009; Goentoro et al., 2009; Goentoro and Kirschner, 2009; Adler and Alon, 2018; Chou, 2018; Xiong et al., 2019; Momin et al., 2020). Signaling pathways can induce different dynamics depending on the activation of the TFs, their potential interaction partners, interacting patterns, and their localizations (Hao et al., 2013). This work explains possible network-level calculations based on specific network motif architecture that facilitates TGFβ-induced EMT.

TGFβ secreted by several cell types is a key external signal of EMT with a complex biphasic function of opposing effects depending on tumor microenvironment (Zavadil and Bottinger, 2005; Massague, 2012; Aykul and Martinez-Hackert, 2016; Derynck et al., 2021; Liu et al., 2021). In premalignant stages, TGFβ promotes cell differentiation, cell cycle arrest, apoptosis, and senescence in epithelial cells (Aykul et al., 2021; Zou et al., 2021). However, during malignancy, TGFβ acts as an inducer of EMT, further promoting cell metastasis (Zavadil and Bottinger, 2005; Massague, 2012; Derynck et al., 2021; Liu et al., 2021). The dynamics of TGFβ-mediated EMT have been extensively investigated using several system-level models (Burger et al., 2017; Celià-Terrassa et al., 2018; Selvaggio et al., 2020; Silveira et al., 2020; Silveira and Mombach, 2020). These investigations have revealed that EMT is not a binary process, and involves many intermediate hybrid states during the transition (Lu et al., 2013b; Tian et al., 2013; Nieto et al., 2016; Xin et al., 2020). These hybrid states were attributed to several epigenetic regulators and post-translational modifications governing the highly interconnected core modules SNAIL:miR-34 and ZEB:miR-200 interacting in a dual negative regulatory fashion (Jolly et al., 2014; Boareto et al., 2016; Jolly et al., 2016; Burger et al., 2017; Jia et al., 2017; Jia et al., 2019). Modeling and analysis of the core regulatory network have contributed tremendously to the understanding of EMT dynamics.

The present work explored the design principles of certain specific regulatory motifs observed in TGFβ signaling that govern EMT. Here, we contemplate the roles of EMT regulators SMAD, GLI, SNAIL, ZEB, and N-cadherin, which are induced by TGFβ organized in the form of coherent type 1 feed-forward loop-like architecture (Figure 2). These C1FFLs may contribute to the temporal establishment of an EMT-based switch. These network motif-like architectures also explain the existence of multiple intermediate states in EMT.

### C1FFLs of TGFβ-induced EMT

Analyses of the C1FFLs (Figure 2) observed within the compiled network (Figure 1) were performed individually. The function of SNAIL (Figure 2A; Figure 3) and N-cadherin (Figures 2B, 4) as noise filters and persistence detectors was revealed through these individual simulations. The function of SNAIL as an integrator of noise buffer has been previously illustrated (Lu et al., 2013b; Lu et al., 2014; Zhang et al., 2018; Bhavani and Palanisamy, 2022). Many experimental studies have also reported the role of TGFβ-induced GLI contributing to sustained activation of SNAIL (Aberger and Altaba, 2014; Zhang et al., 2018). This work demonstrated that the activity of SNAIL depends on SMAD and GLI induced by TGFβ in a C1FFL regulatory manner (Figure 2A). In addition, SNAIL has been shown to directly regulate the expression of the EMT transcription factor ZEB (Peinado et al., 2007; Gregory et al., 2011). Studies have also highlighted the role of ZEB as a master regulator of cell fate decision-making during EMT (Burk et al., 2008; Brabletz and Brabletz, 2010; Lu et al., 2014). SNAIL was found to regulate the activity of the EMT marker N-cadherin (Thiery et al., 2009; Wang et al., 2015; Cao et al., 2019). Cadherins are the cell surface molecules involved in the adhesion mechanism. During the developmental process, cadherins play a major role in regulating cell–cell adhesion and in modulating morphogenetic and differentiation processes (Vestweber, 2015). Cadherin also enhances phenotypic change that promotes tumor cell migration and motility (Loh et al., 2019). Our observations are in agreement with these experimental findings. The present work illustrates that the regulation between the EMT core transcription factors SNAIL and ZEB were observed to occur in a C1FFL manner, while regulating the activity of the mesenchymal marker N-cadherin (Figure 2B). Thus, these C1FFLs, analyzed as individual modules, contribute towards executing EMT.

### Hypermotif regulating N-cadherin

Cancer metastasis driven by EMT involves complex circuits composed of several network motifs with varying topology. One such module was identified between the C1FFLs (Figures 2A, B) organized in a layered cascade/hypermotif (Figure 2C). The present study explored the effect of the two C1FFLs combined through SNAIL, which functions as a ‘signal integrator.’ The function of SNAIL as an integrator of upstream signals has been illustrated previously, in both experimental and system-level studies (Lu et al., 2013b; Wang et al., 2013; Lu et al., 2014; Zhang et al., 2018; Bhavani and Palanisamy, 2022). Dynamic modeling and analysis of the hypermotif (Figure 2C) have highlighted the emergent behavior of EMT regulators SMAD, GLI, SNAIL, and ZEB by influencing N-cadherin expression, one of the key decision-makers in EMT (Figure 2C). The simulations have shown temporal regulation of the individual regulators, along with their emergent properties, such as noise filter, persistent detection in regulating the activity of N-cadherin. The response time of these individual regulators may correlate with their functional role during phenotypic transition, which needs further experimental validation.

EMT is one of the crucial cellular functions. Type I and type II EMT are associated mainly with the physiological functions of a cell, such as embryogenesis, organ development, tissue development, and organ fibrosis, whereas type III EMT is associated with the pathophysiological functions of a cell, such as the progression of neoplasia and metastasis (Kalluri and Weinberg, 2009). However, the emergence of type III EMT as a function of intertwined environmental cues and epigenetic factors is much debated and unclear. Although many underlying subnetworks associated with EMT in tumorigenesis have been identified, the mechanisms and design principles that enable robustness in EMT are not fully understood (Watanabe et al., 2019; Hari et al., 2020; Hebbar et al., 2022). This work illustrates that the hypermotif may contribute to the observed robust behavior and existence of multiple intermediate states during EMT. Such architecture may also contribute to the hybrid states with the co-existence of epithelial and mesenchymal phenotypes. Thus, layered C1FFLs (also known as hypermotifs) may process signaling information towards executing EMT in cancer.

Epithelial to mesenchymal transition (ON led end phenotype) and mesenchymal to epithelial transition (OFF led end phenotype) are morphologically and physiologically different events that are temporally and spatially separated. This work explored only the ON step of the signal processing guiding EMT (also known as epithelial to mesenchymal phenotypic switch) during cancer metastasis. This work hints at a possible role of FFLs apart from the known dual negative regulatory motifs in TGFβ-induced EMT. Core EMT transcription factors SNAIL and ZEB are part of both the FFLs and double-negative feedback loops.

A multitude of cellular functions, such as growth and differentiation, are mediated by genetic circuits in layers. FFLs are often organized through various architectural principles with distinct information processing functions (Kaplan et al., 2008; Cohen and Rosner, 2009; Chepyala et al., 2016; Alon, 2019; Chakravarty and Csikász-Nagy, 2021; Pieters et al., 2021; Baumann et al., 2022). It has been recognized that even the same network motifs emerge in several contexts, the way they are organized provides distinct features (Adler and Medzhitov, 2022). The motifs and hypermotifs analyzed as part of this work revealed the information processing functions of the complex interaction network of TGFβ-induced EMT in metastatic cancer progression. The results of this study offer new insights into the emergence of type III EMT during cancer metastasis.

### Information processing by layered C1FFLs

Biological networks comprise webs of biomolecular interactions and reactions connected through motifs in layers. Several studies have related individual motif functions to the larger complex network-based regulation of cellular behavior (Alon, 2007; Ferrell and Ha, 2014; Alon, 2019; Adler and Medzhitov, 2022). Motivated by such analyses, a three-layered C1FFL hypermotif was postulated and explored for its possible dynamic properties. This more topologically complex structure executed temporal regulation of the individual regulators in all the layers, filtered noise, and functioned as a persistence and coincidence signal detector. Thus, deciphering this network architecture has provided some insights and clues. Such networks, although hypothesized in this study, may exist and remain unexplored in real biological networks.

### Sensitivity analysis identifies key influencers of EMT

Sensitivity analysis methods are classified into local or global methods (Perumal and Gunawan, 2011; Razavi and Gupta, 2015). Local methods employ a one-at-a-time approach in which model responses are analyzed while one parameter at a time is varied (Chen et al., 2018; Jolly et al., 2018; Faro et al., 2019; Storey et al., 2020; Subbalakshmi et al., 2021; Dela et al., 2022; Ryan et al., 2022). In contrast, global methods explore model responses by varying all the parameters at the same time within a range of uncertainty (Dela et al., 2022). The current study utilized a local sensitivity analysis to explore the performance of the model when individual parameters were perturbed within a small range from the nominal set of parameters. Increased activity of SMAD or SNAIL in the C1FFLs (Figures 2A, B) resulted in early activation of SNAIL or N-cadherin, while curtailing their levels resulted in delayed activation (Figures 7A, B). Increased activity of SMAD, GLI, and SNAIL in the hypermotif regulating N-cadherin (Figure 2C) resulted in early activation of N-cadherin. Curtailed activities of those transcription factors resulted in delayed activation of N-cadherin, suggesting that the levels of these regulators play an important role in initiating EMT or hybrid EMT phenotypes (Jia et al., 2015). Parameter sensitivity analysis of a hypothesized hypermotif suggested that, in a layered architecture (Figure 6A), T_on_ of the end response regulator Z_3_ is sensitive to changes in the parameter variations of regulators that are involved in the foremost layer of the hypermotif. T_on_ was observed to have a robust response to the parameter variations associated with the subsequent layers of the hypermotif (Figure 7D). The local parameter sensitivity analysis revealed that the activation time (T_on_) of the end response regulator was altered in response to variations in individual parameters; however, the overall responses of the motifs and hypermotifs were preserved.

In summary, this work elucidates an alternative view of TGFβ-induced EMT and metastasis driven by a combination of feed-forward loop-like architecture. Modeling and analysis of these motifs indicated significant roles of such network architecture in executing EMT in cancer metastasis. Exploring biological networks for such modular biological units and functions experimentally will lead to deeper understanding of cancer. It is such modules which are executing deleterious processes such as metastatic cancer progression; hence modules can be targeted instead of individual regulators for drug development and treatment.

## Methods

### Networks, motifs, and hypermotifs

EMT can be induced by a plethora of stimuli and confers malignant properties that increase the invasiveness of cancer cells during metastasis. TGFβ signaling stimulates the expression of several regulators and is a potent inducer of EMT. Therefore, in order to explore the role of various regulators induced by TGFβ in EMT, a network was assembled through an extensive literature survey (Figure 1). Information on the various regulators involved in TGFβ-induced EMT is detailed in Supplementary Table S5 of Supplementary File S1. The modeling studies adhered to reduced nomenclature to avoid confusion and ensure clarity. The assembled network comprised various regulators, including SMAD, GLI, SNAIL, ZEB, N-cadherin, and E-cadherin, which are regulated through both TGFβ-induced SMAD-dependent and SMAD-independent pathways. These regulators were interconnected through several network motifs and hypermotif-like architectures (Figure 2), which play crucial roles in moderating the process of EMT.

### Continuous dynamic modeling and simulations

Dynamic ordinary differential equation (ODE)-based modeling and simulations are useful in the quantification of emergent properties of complex biological network structures (Kreutz, 2020). To explore the emergent dynamics of the motifs (Figures 2A, B) and the hypermotifs (Figure 2C) within the compiled network (Figure 1), ODE-based models were developed. Model simulations and analyses were performed to explore the information processing roles of these motifs and hypermotifs that are regulated by TGFβ-induced EMT.

### Modeling C1FFLs

Several models of C1FFLs have been explored, and their system-level properties have been illustrated (Mangan and Alon, 2003; Ghosh et al., 2005; Kalir et al., 2005; Chepyala et al., 2016; Chakravarty and Csikász-Nagy, 2021; Pieters et al., 2021; Bhavani and Palanisamy, 2022). Similar approaches were followed to characterize the role of the C1FFLs shown in Figure 2 in moderating the process of EMT in cancer progression. After an input stimulus S from TGFβ, system-level properties were explored.

The motifs shown in Figure 2 regulating SNAIL and N- cadherin, respectively, were represented in terms of the ODE-based equations. Mass action, and Hill equation-based model formulations were utilized to represent the dynamic Michaelis–Menten kinetics regulation of individual regulators. Both AND and OR logic-like regulations were independently explored for stimulus-dependent regulation of SNAIL and N-cadherin. Combinations of continuous and pulsatile inputs were provided as the stimuli.

### C1FFL model of SNAIL regulation

$$\frac{d\left[SMAD\right]}{dt}=S*S_{1}\left(t\right)-kd_{1}*\left[SMAD\right],$$ (1)

$$\frac{d\left[GLI\right]}{dt}=\frac{k_{1}*{\left[SMAD\right]}^{n}}{Km_{1}^{n}+{\left[SMAD\right]}^{n}}-kd_{2}*\left[GLI\right],$$ (2)

$$\frac{d\left[SNAIL\right]}{dt}=P_{Z}\left(GLI,k_{2};\;SMAD,k_{3}\right)-kd_{3}*\left[SNAIL\right].$$ (3)

The function for SNAIL activation $P_{Z}\left(GLI,k_{2};\;SMAD,k_{3}\right)$ in an AND logic gate-like manner is represented as $\frac{k_{2}*{\left[GLI\right]}^{n}}{Km_{2}^{n}+{\left[GLI\right]}^{n}}*\frac{k_{3}*{\left[SMAD\right]}^{n}}{Km_{3}^{n}+{\left[SMAD\right]}^{n}}$ and in an OR logic gate is written as $\frac{k_{2}*{\left[GLI\right]}^{n}}{Km_{2}^{n}+{\left[GLI\right]}^{n}}+\frac{k_{3}*{\left[SMAD\right]}^{n}}{Km_{3}^{n}+{\left[SMAD\right]}^{n}}$ .

### C1FFL model for N-cadherin expression

$$\frac{d\left[SNAIL\right]}{dt}=S*S_{1}\left(t\right)-kd_{4}*\left[SNAIL\right],$$ (4)

$$\frac{d\left[ZEB\right]}{dt}=\frac{k_{4}*{\left[SNAIL\right]}^{n}}{Km_{4}^{n}+{\left[SNAIL\right]}^{n}}-kd_{5}*\left[ZEB\right],$$ (5)

$$\frac{d\left[N-Cadherin\right]}{dt}=S_{Z}\left(ZEB,k_{5};\;SNAIL,k_{6}\right)-kd_{6}*\left[N-Cadherin\right].$$ (6)

The function for N-cadherin regulation $S_{Z}\left(ZEB,k_{5};\;SNAIL,k_{6}\right)$ in an AND logic gate-like manner is represented as $\frac{k_{5}*{\left[ZEB\right]}^{n}}{Km_{5}^{n}+{\left[ZEB\right]}^{n}}*\frac{k_{6}*{\left[SNAIL\right]}^{n}}{Km_{6}^{n}+{\left[SNAIL\right]}^{n}}$ and in an OR logic-like gate is written as $\frac{k_{5}*{\left[ZEB\right]}^{n}}{Km_{5}^{n}+{\left[ZEB\right]}^{n}}+\frac{k_{6}*{\left[SNAIL\right]}^{n}}{Km_{6}^{n}+{\left[SNAIL\right]}^{n}}$ .

### Two-layered C1FFL model of N-cadherin

$$\frac{d\left[SMAD\right]}{dt}=S*S_{1}\left(t\right)-kd_{1}*\left[SMAD\right],$$ (7)

$$\frac{d\left[GLI\right]}{dt}=\frac{k_{1}*{\left[SMAD\right]}^{n}}{Km_{1}^{n}+{\left[SMAD\right]}^{n}}-kd_{2}*\left[GLI\right],$$ (8)

$$\frac{d\left[SNAIL\right]}{dt}=Q_{Z}\left(GLI,k_{2};\;SMAD,k_{3}\right)-kd_{3}*\left[SNAIL\right],$$ (9)

$$\frac{d\left[ZEB\right]}{dt}=\frac{k_{4}*{\left[SNAIL\right]}^{n}}{Km_{4}^{n}+{\left[SNAIL\right]}^{n}}-kd_{4}*\left[ZEB\right],$$ (10)

$$\frac{d\left[N-Cadherin\right]}{dt}=R_{Z}\left(ZEB,k_{5};\;SNAIL,k_{6}\right)-kd_{5}*\left[N-Cadherin\right].$$ (11)

$Q_{Z}\left(GLI,k_{2};\;SMAD,k_{3}\right)$ and $R_{Z}\left(ZEB,k_{5};\;SNAIL,k_{6}\right)$ are functions for SNAIL and N-cadherin in an AND logic gate-like manner and are represented as $\frac{K_{2}*{\left[GLI\right]}^{n}}{Km_{2}^{n}+{\left[GLI\right]}^{n}}*\frac{K_{3}*{\left[SMAD\right]}^{n}}{Km_{3}^{n}+{\left[SMAD\right]}^{n}}$ and $\frac{k_{5}*{\left[ZEB\right]}^{n}}{Km_{5}^{n}+{\left[ZEB\right]}^{n}}*\frac{k_{6}*{\left[SNAIL\right]}^{n}}{Km_{6}^{n}+{\left[SNAIL\right]}^{n}}$ , respectively.

### Hypothetical three-layered C1FFL model

The hypothetical three-layered C1FFL network shown in Figure 6A was represented in terms of the ODE-based equations. Mass action, Michaelis–Menten kinetics, and Hill equation-based model formulations were utilized to represent the dynamic regulation of individual regulators.

$$\frac{d\left[X_{1}\right]}{dt}=S_{x}*S_{1}\left(t\right)-kd_{1}*\left[X_{1}\right],$$ (12)

$$\frac{d\left[Y_{1}\right]}{dt}=S_{y}*S_{2}\left(t\right)+\frac{k_{1}*{\left[X_{1}\right]}^{n}}{Km_{1}^{n}+{\left[X_{1}\right]}^{n}}-kd_{2}*\left[Y_{1}\right],$$ (13)

$$\frac{d\left[Z_{1}\right]}{dt}=A\left(Y_{1},k_{2};\;X_{1},k_{3}\right)-kd_{3}*\left[Z_{1}\right],$$ (14)

$$\frac{d\left[Y_{2}\right]}{dt}=\frac{k_{4}*{\left[Z_{1}\right]}^{n}}{Km_{4}^{n}+{\left[Z_{1}\right]}^{n}}-kd_{4}*\left[Y_{2}\right],$$ (15)

$$\frac{d\left[Z_{2}\right]}{dt}=B\left(Y_{2},k_{5};\;Z_{1},k_{6}\right)-kd_{5}*\left[Z_{2}\right],$$ (16)

$$\frac{d\left[Y_{3}\right]}{dt}=\frac{k_{7}*{\left[Z_{2}\right]}^{n}}{Km_{7}^{n}+{\left[Z_{2}\right]}^{n}}-kd_{6}*\left[Y_{3}\right],$$ (17)

$$\frac{d\left[Z_{3}\right]}{dt}=C\left(Y_{3},k_{8};\;Z_{2},k_{9}\right)-kd_{7}*\left[Z_{3}\right],$$ (18)

$A\left(Y_{1},k_{2};\;X_{1},k_{3}\right),B\left(Y_{2},k_{5};\;Z_{1},k_{6}\right),and\;C\left(Y_{3},k_{8};\;Z_{2},k_{9}\right)$ are the regulatory functions for Z_1_, Z_2_, and Z_3_ in an AND logic gate-like manner and are represented as $\frac{k_{2}*{\left[Y_{1}\right]}^{n}}{Km_{2}^{n}+{\left[Y_{1}\right]}^{n}}*\frac{k_{3}*{\left[X_{1}\right]}^{n}}{Km_{3}^{n}+{\left[X_{1}\right]}^{n}}$ , $\frac{k_{5}*{\left[Y_{2}\right]}^{n}}{Km_{5}^{n}+{\left[Y_{2}\right]}^{n}}*\frac{k_{6}*{\left[Z_{1}\right]}^{n}}{Km_{6}^{n}+{\left[Z_{1}\right]}^{n}}$ , and $\frac{k_{8}*{\left[Y_{3}\right]}^{n}}{Km_{8}^{n}+{\left[Y_{3}\right]}^{n}}*\frac{k_{9}*{\left[Z_{2}\right]}^{n}}{Km_{9}^{n}+{\left[Z_{2}\right]}^{n}}$ , respectively.

All the ODE models developed were simulated using the *ode23s* solver of MATLAB R2022a. The associated parameters are tabulated in Supplementary File 1. These parameters were chosen from previous models (Lu et al., 2013b; Tian et al., 2013; Zhang et al., 2018).

### Parameter sensitivity analysis

Local parameter sensitivity analysis of the motifs and hypermotifs was performed to investigate the model responses for small perturbations in parameters. The model parameters listed in Supplementary Table S1–S4 were varied one at a time within a ±30% (intervals of ±10%) range from their nominal value. The sensitivity of a model is measured by assessing how changes in the parameters affect the time for activation of the end response regulators of the motifs and the hypermotif after the stimulus is provided, which is indicated as T_on_. T_on_ for activating EMT regulators SNAIL and N-cadherin in individual motifs were 2.1 and 2.4 time units, respectively. T_on_ for activating N-cadherin in the hypermotif was 7.6 time units and 11.3 time units for the hypothetical motif. The time required for the activation of an end regulator (T_on_) when the individual parameters were varied (within a ±30% range) was assessed and compared with the T_on_ value obtained for the nominal parameter set.

## Data Availability

The original contributions presented in the study are included in the article/Supplementary Material; further inquiries can be directed to the corresponding author.
