# Supplementary material for: Network motifs and hypermotifs in TGFβ-induced epithelial to mesenchymal transition and metastasis
Source: Front Syst Biol. 2023 Mar 3;3:1099951. doi: 10.3389/fsysb.2023.1099951 (PMC12342032; doi:10.3389/fsysb.2023.1099951)
Supplement: Supplementary file 1 [file DataSheet1.pdf]

Supplementary information for

Network Motifs and hypermotif in TGFβ induced Epithelial to Mesenchymal Transition and Metastasis

Gottumukkala Sai Bhavani[1], Anbumathi Palanisamy [2]*,

[1] Research Scholar, Department of Biotechnology, NIT Warangal, India.

[2] Assistant Professor, Department of Biotechnology, NIT Warangal, India.

*Correspondence:
Anbumathi Palanisamy

anbu@nitw.ac.in

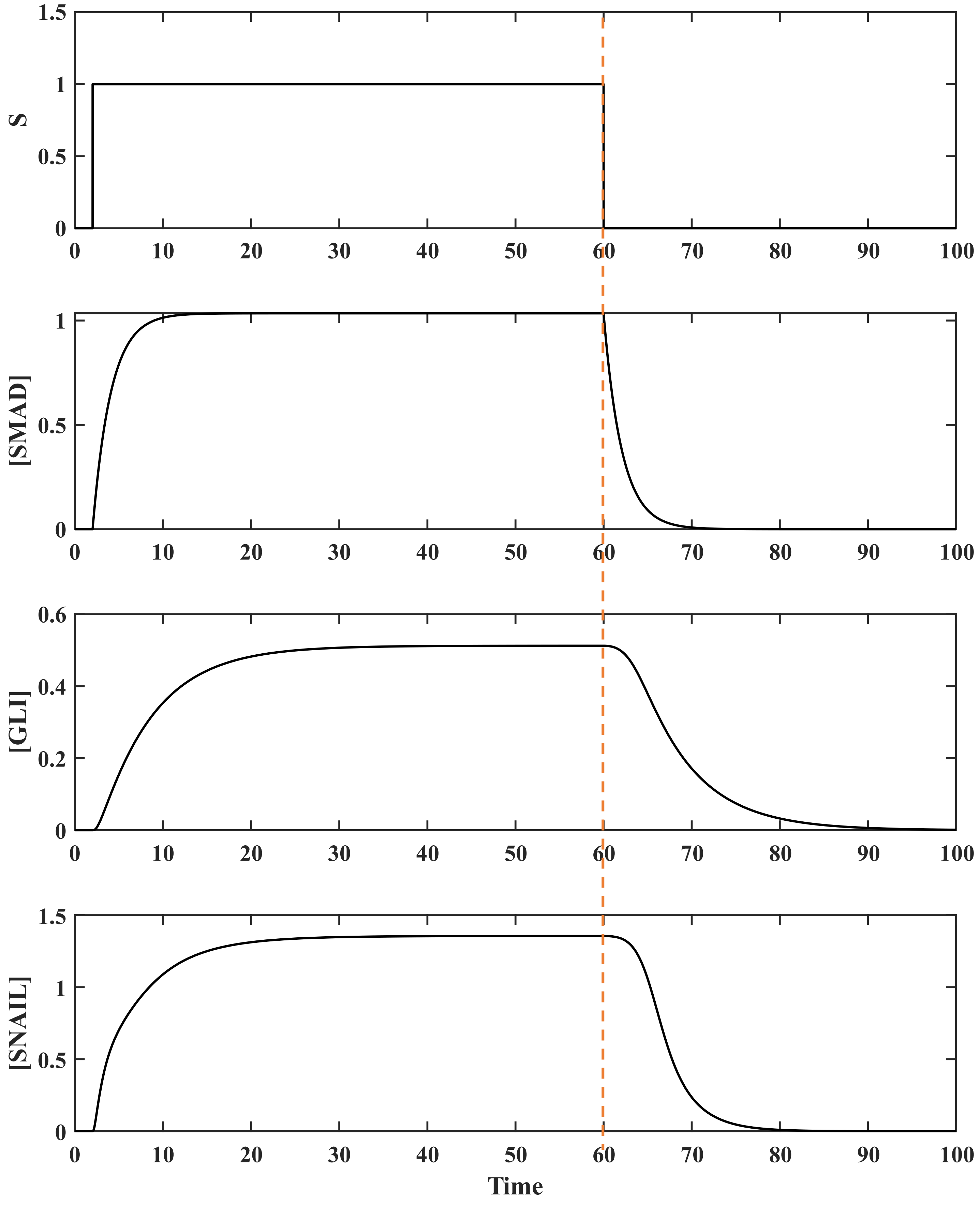

Figure S1. Dynamics of TGF$\boldsymbol{\beta}$ induced activation of SNAIL in a coherent type1 feed forward regulation directly by SMAD and indirectly through GLI with respect to OR regulatory logic for input stimulus S. A delay was observed in the SNAIL repression when the stimulus was withdrawn.

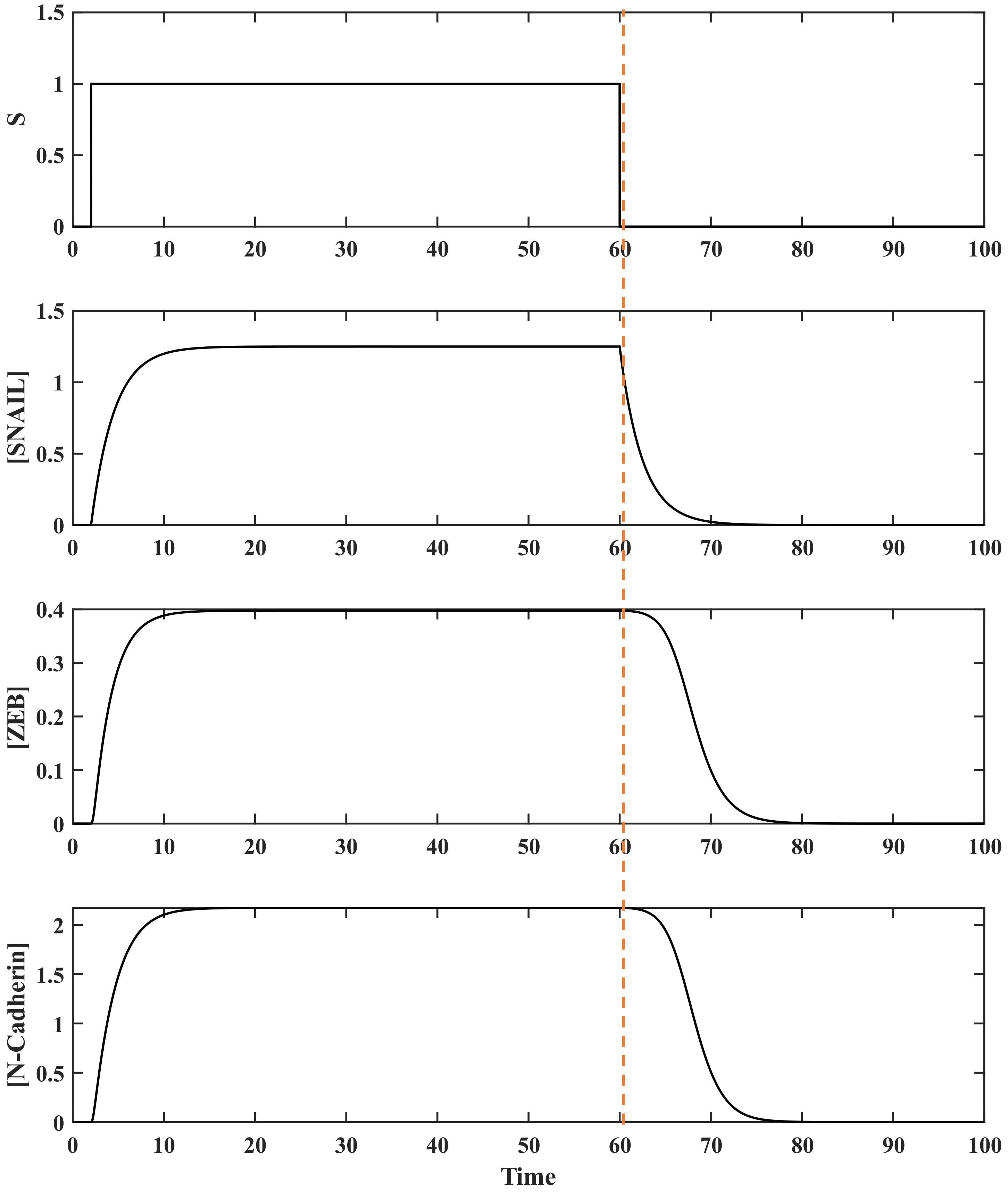

Figure S2. Regulation of Mesenchymal gene N-Cadherin by SNAIL, ZEB induced by TGF$\boldsymbol{\beta}$ in a C1FFL manner with respect to AND regulatory logic for input stimulus S. A delay was observed in the N-Cadherin repression when the stimulus was withdrawn.

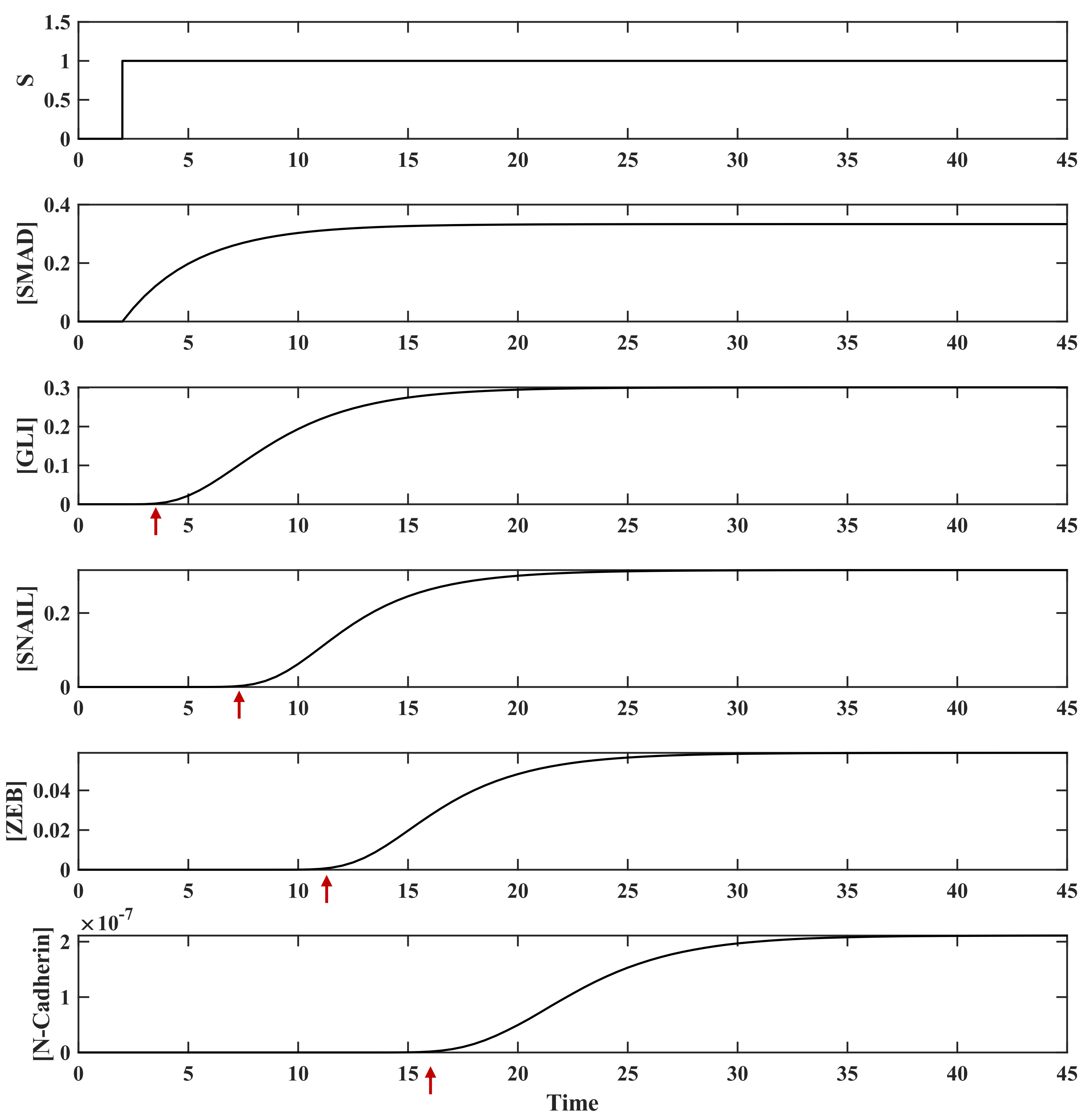

Figure S3. Hypermotif regulating N-Cadherin expression. Dynamics of the cascade for increasing Hill coefficient (n), i.e., n=4. For larger ‘n’ the cascade was observed have a delayed response in regulating the expression of N-Cadherin.

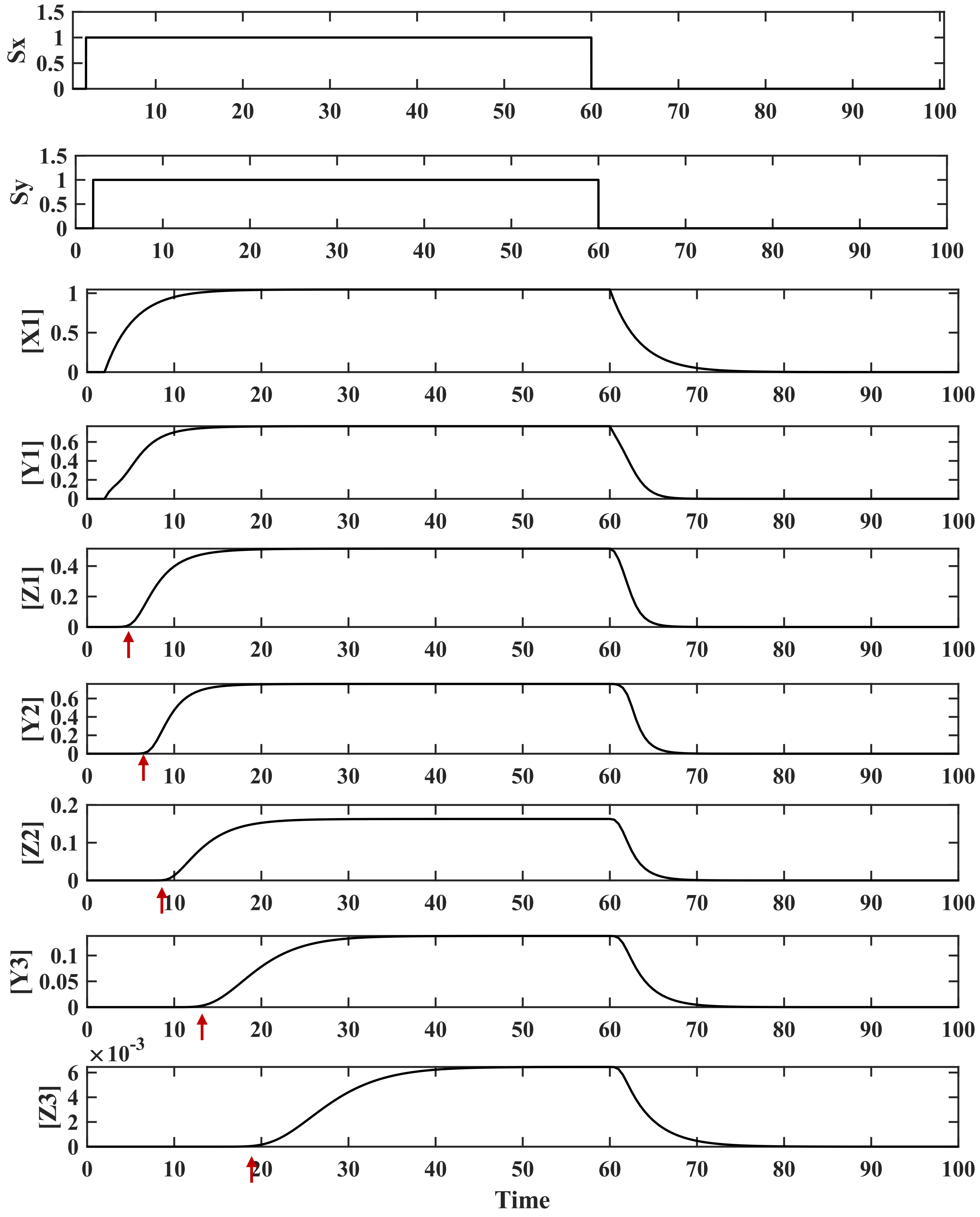

Figure S4. Dynamics of hypothesized combination of three C1FFLs (hypermotif) in layer for larger Hill coefficient n = 4. It was observed that the network with higher Hill coefficient generates a delayed temporal program of regulators.

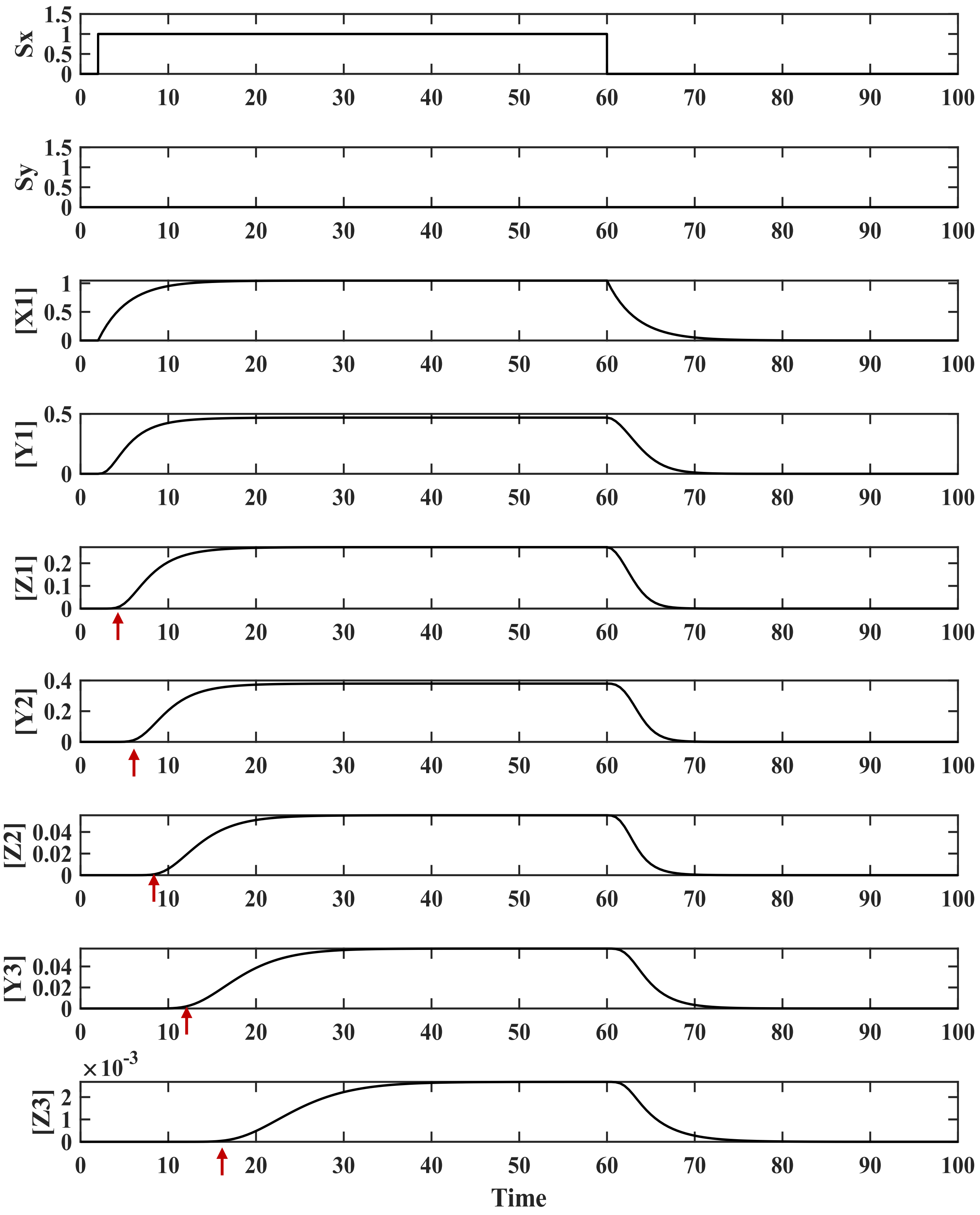

Figure S5. Dynamic of the hypothesized 3 layered network (hypermotif) when the stimulus S_y was turned OFF. In this scenario even if the S_y is OFF Z_3 achieves maximum activity through Z_1 in Z_2 by the activation of Y1 by X1 through S_x.

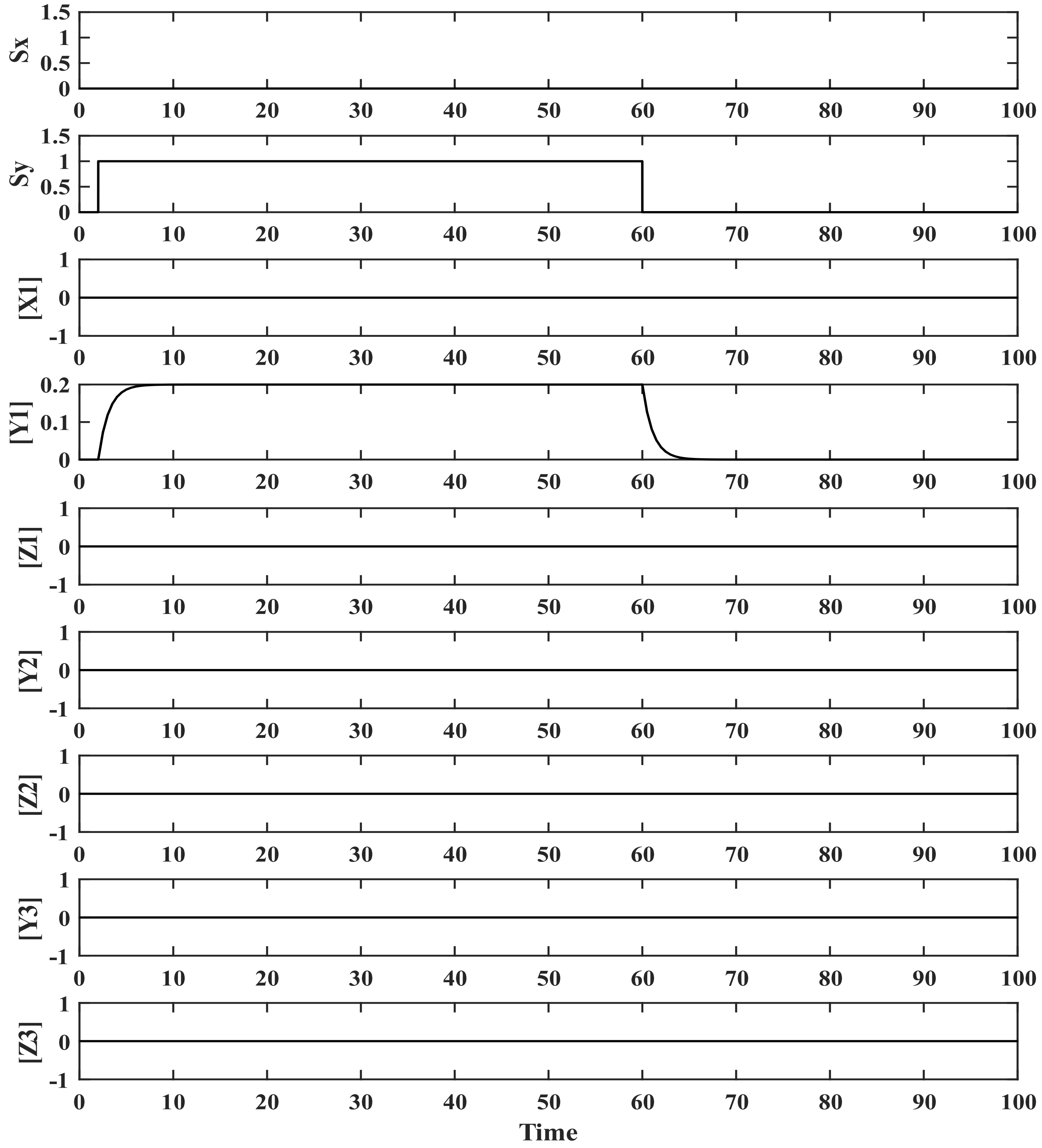

Figure S6. Dynamic of the hypothesized 3 layered network (hypermotif) when the stimulus S_x was turned OFF. The absence of S_x do not alleviate any response

Table S1. Parameters for the Dynamic Modelling and simulation of C1FFL regulating SNAIL

Description	AND Logic	OR Logic
TGF-β dependent activation of SMAD complex (S)	0.2	0.5
Production rate of SMAD dependent GLI (k1)	0.09	0.09
Michaelis Menten constant for SMAD dependent GLI production (Km1)	0.25	0.25
Production rate of GLI dependent SNAIL (k2)	0.9	0.9
Michaelis Menten constant for GLI dependent SNAIL production (Km2)	0.47	0.47
Production rate of SMAD dependent SNAIL (k3)	0.9	0.6
Michaelis Menten constant for SMAD dependent SNAIL production (Km3)	0.5	0.09
Degradation rate of SMAD (kd1)	0.4833	0.4833
Degradation rate of GLI (kd2)	0.166	0.166
Degradation rate of SNAIL (kd3)	0.8	0.5
Hill Coefficient (n)	2	2

Table S2. Parameters for the Dynamic Modelling and simulation of C1FFL regulating N-Cadherin

Description	AND Logic	OR Logic
TGF-β Dependent activation Of SNAIL (S)	0.06	0.5
Production rate of SNAIL dependent ZEB (k4)	0.2	0.2
Michaelis Menten constant For SNAIL dependent ZEB Production (Km4)	0.1	0.1
Production rate Of ZEB dependent N-Cadherin (k5)	0.8	0.8
Michaelis Menten constant for ZEB dependent N-Cadherin production (Km5)	0.5	0.5
Production rate of SNAIL dependent N-Cadherin (k6)	1	1
Michaelis Menten constant for SNAIL dependent N-Cadherin production (Km6)	0.3	0.1
Degradation rate Of SNAIL (kd4)	0.4	0.4
Degradation rate Of ZEB (kd5)	0.5	0.5
Degradation rate of N-Cadherin (kd6)	0.6	0.6
Hill Coefficient (n)	2	2

Table S3. Parameters for the Dynamic Modelling of Combination of Two C1FFLs Regulating N-Cadherin

**Description**	**Parameters**
TGF-β dependent activation of SMAD complex (S)	0.1
Degradation rate SMAD complex (kd1)	0.3
Degradation rate GLI (kd2)	0.6
Degradation rate SNAIL (kd3)	0.9
Degradation rate ZEB (kd4)	1.66
Degradation rate N-Cadherin (kd5)	0.8
Production rate of SMAD complex dependent GLI (k1)	0.4
Production rate of GLI dependent SNAIL (k2)	0.85
Production rate of SMAD complex dependent SNAIL (k3)	0.74
Production rate of SNAIL dependent ZEB (k4)	0.5
Production rate of ZEB dependent N-Cadherin (k5)	0.7
Production rate of SNAIL dependent N-Cadherin (k6)	0.7
Michaelis Menten constant for SMAD complex dependent GLI production (Km1)	0.35
Michaelis Menten constant for GLI dependent SNAIL production (Km2)	0.25
Michaelis Menten constant for SMAD complex dependent SNAIL production (Km3)	0.28
Michaelis Menten constant for SNAIL dependent ZEB Production (Km4)	0.45
Michaelis Menten constant For ZEB dependent N-Cadherin Production (Km5)	0.9
Michaelis Menten constant For SNAIL dependent N-Cadherin production (Km6)	0.85
Hill coefficient	2,4

Table S4. Parameters for the Dynamic Modelling of Hypothesised Combination of Three C1FFLs in Layer

**Description**	**Parameters**
S_x Dependent activation of X_1	0.314
S_y dependent activation of Y_1	0.18
Degradation rate of X_1 (kd1)	0.3
Degradation rate of Y_1 (kd2)	0.9
Degradation rate of Z_1 (kd3)	0.86
Degradation rate of Y_2 (kd4)	0.78
Degradation rate of Z_2 (kd5)	0.6
Degradation rate of Y_3 (kd6)	0.4
Degradation rate of Z_3 (kd7)	0.3
Production rate of X_1 dependent Y_1 (k1)	0.6
Production rate of Y_1 dependent Z_1 (k2)	0.85
Production rate of X_1 dependent Z_1 (k3)	0.75
Production rate of Z_1 dependent Y_2 (k4)	0.66
Production rate of Y_2 dependent Z_2 (k5)	0.83
Production rate of Z_1 dependent Z_2 (k6)	0.89
Production rate of Z_2 dependent Y_3 (k7)	0.69
Production rate of Y_3 dependent Z_3 (k8)	0.87
Production rate of Z_2 dependent Z_3 (k9)	0.92
Michaelis Menten constant for X_1 dependent Y_1 production (Km1)	0.68
Michaelis Menten constant for Y_1 dependent Z_1 production (Km2)	0.4
Michaelis Menten constant for X_1 dependent Z_1 production (Km3)	0.8
Michaelis Menten constant for Z_1 dependent Y_2 production (Km4)	0.3
Michaelis Menten constant for Y_2 dependent Z_2 production (Km5)	0.43
Michaelis Menten constant for Z_1 dependent Z_2 production (Km6)	0.8
Michaelis Menten constant for Z_2 dependent Y_3 production (Km7)	0.3
Michaelis Menten constant for Y_3 dependent Z_3 production (Km8)	0.19
Michaelis Menten constant for Z_2 dependent Z_3 production (Km9)	0.5
Hill coefficient	2,4

Table S5. TGFβ induced regulators considered for the regulatory network

Model Representation	Biological Nomenclature	References
R-SMAD	SMAD 2/3	(Attisano and Wrana, 1998, Derynck and Zhang, 2003, Zhang et al., 2014, Tu et al., 2019, Sundqvist et al., 2012)
Co-SMAD	SMAD 4	(Attisano and Wrana, 1998, Derynck and Zhang, 2003)
SNAIL	SNAI1, SNAI2	(Tian et al., 2013, Lu et al., 2013, Peinado et al., 2007, Lai et al., 2016, He et al., 2018, Li and Balazsi, 2018, Jolly et al., 2016, Hemavathy et al., 2000)
ZEB	ZEB1, ZEB2	(Tian et al., 2013, Lu et al., 2013, Peinado et al., 2007, Lai et al., 2016, He et al., 2018, Li and Balazsi, 2018, Jolly et al., 2016, Gheldof et al., 2012)
GLI	GLI1	(Zhang et al., 2018, Maheshwari et al., 2022, Zhang et al., 2016, Fröhlich et al., 2015)
N-Cadherin	CDH2	(Tian et al., 2013, Xing and Tian, 2019, Loh et al., 2019)
E-Cadherin	CDH1	(Tian et al., 2013, Xing and Tian, 2019, Tripathi et al., 2021, Xin et al., 2020)

References:

ATTISANO, L. & WRANA, J. L. 1998. Mads and Smads in TGF beta signalling. *Curr Opin Cell Biol*, 10, 188-94.

DERYNCK, R. & ZHANG, Y. E. 2003. Smad-dependent and Smad-independent pathways in TGF-beta family signalling. *Nature*, 425, 577-84.

FRÖHLICH, H., BAHAMONDEZ, G., GÖTSCHEL, F. & KORF, U. 2015. Dynamic Bayesian Network Modeling of the Interplay between EGFR and Hedgehog Signaling. *PLOS ONE*, 10, e0142646.

GHELDOF, A., HULPIAU, P., VAN ROY, F., DE CRAENE, B. & BERX, G. 2012. Evolutionary functional analysis and molecular regulation of the ZEB transcription factors. *Cellular and Molecular Life Sciences*, 69, 2527-2541.

HE, P., QIU, K. & JIA, Y. 2018. Modeling of mesenchymal hybrid epithelial state and phenotypic transitions in EMT and MET processes of cancer cells. *Sci Rep*, 8, 14323.

HEMAVATHY, K., ASHRAF, S. I. & IP, Y. T. 2000. Snail/slug family of repressors: slowly going into the fast lane of development and cancer. *Gene*, 257, 1-12.

JOLLY, M. K., TRIPATHI, S. C., JIA, D., MOONEY, S. M., CELIKTAS, M., HANASH, S. M., MANI, S. A., PIENTA, K. J., BEN-JACOB, E. & LEVINE, H. 2016. Stability of the hybrid epithelial/mesenchymal phenotype. *Oncotarget*, 7, 27067-84.

LAI, X., WOLKENHAUER, O. & VERA, J. 2016. Understanding microRNA-mediated gene regulatory networks through mathematical modelling. *Nucleic Acids Res*, 44, 6019-35.

LI, C. & BALAZSI, G. 2018. A landscape view on the interplay between EMT and cancer metastasis. *NPJ Syst Biol Appl*, 4, 34.

LOH, C.-Y., CHAI, J., TANG, T., WONG, W., SETHI, G., SHANMUGAM, M., CHONG, P. & LOOI, C. 2019. The E-Cadherin and N-Cadherin Switch in Epithelial-to-Mesenchymal Transition: Signaling, Therapeutic Implications, and Challenges. *Cells*, 8, 1118.

LU, M., JOLLY, M. K., LEVINE, H., ONUCHIC, J. N. & BEN-JACOB, E. 2013. MicroRNA-based regulation of epithelial-hybrid-mesenchymal fate determination. *Proc Natl Acad Sci U S A*, 110, 18144-9.

MAHESHWARI, P., ASSMANN, S. M. & ALBERT, R. 2022. Inference of a Boolean Network From Causal Logic Implications. *Frontiers in Genetics*, 13.

PEINADO, H., OLMEDA, D. & CANO, A. 2007. Snail, Zeb and bHLH factors in tumour progression: an alliance against the epithelial phenotype? *Nat Rev Cancer*, 7, 415-28.

SUNDQVIST, A., TEN DIJKE, P. & VAN DAM, H. 2012. Key signaling nodes in mammary gland development and cancer: Smad signal integration in epithelial cell plasticity. *Breast Cancer Research*, 14, 204.

TIAN, X. J., ZHANG, H. & XING, J. 2013. Coupled reversible and irreversible bistable switches underlying TGFbeta-induced epithelial to mesenchymal transition. *Biophys J*, 105, 1079-89.

TRIPATHI, S., XING, J., LEVINE, H. & JOLLY, M. K. 2021. Mathematical modeling of plasticity and heterogeneity in EMT. *The Epithelial-to Mesenchymal Transition*. Springer.

TU, HUANG, HUANG, LUO & YAN 2019. Contextual Regulation of TGF-β Signaling in Liver Cancer. *Cells*, 8, 1235.

XIN, Y., CUMMINS, B. & GEDEON, T. 2020. Multistability in the epithelial-mesenchymal transition network. *BMC Bioinformatics*, 21, 71.

XING, J. & TIAN, X.-J. 2019. Investigating epithelial-to-mesenchymal transition with integrated computational and experimental approaches. *Physical Biology*, 16, 031001.

ZHANG, J., TIAN, X.-J., CHEN, Y.-J., WANG, W., WATKINS, S. & XING, J. 2018. Pathway crosstalk enables cells to interpret TGF-β duration. *npj Systems Biology and Applications*, 4.

ZHANG, J., TIAN, X.-J. & XING, J. 2016. Signal Transduction Pathways of EMT Induced by TGF-β, SHH, and WNT and Their Crosstalks. *Journal of Clinical Medicine*, 5, 41.

ZHANG, J., ZHANG, X., XIE, F., ZHANG, Z., VAN DAM, H., ZHANG, L. & ZHOU, F. 2014. The regulation of TGF-β/SMAD signaling by protein deubiquitination. *Protein & Cell*, 5, 503-517.
